# Personality Assessment Inventory in Fibromyalgia: Links to Functional, Physical–Somatic, and Emotional Impact

**DOI:** 10.3390/ejihpe15080149

**Published:** 2025-08-01

**Authors:** Andrea Doreste, Jesus Pujol, Eva Penelo, Víctor Pérez, Laura Blanco-Hinojo, Gerard Martínez-Vilavella, Fabiola Ojeda, Jordi Monfort, Joan Deus

**Affiliations:** 1 Department of Clinical and Health Psychology, Universitat Autònoma de Barcelona, 08193 Barcelona, Spain; 2 MRI Research Unit, Radiology Department, Hospital del Mar, 08003 Barcelona, Spain; 21404jpn@comb.cat (J.P.); laura.blanco02@gmail.com (L.B.-H.); g.martinezvilavella@gmail.com (G.M.-V.); 3 Departament de Psicobiologia I de Metodologia de les Ciències de la Salut, Universitat Autònoma de Barcelona, 08193 Barcelona, Spain; eva.penelo@uab.cat; 4 Neurociences Research Unit, IMIM—Institut Hospital del Mar d’Investigacions Mèdiques, 08003 Barcelona, Spain; vperezsola@psmar.cat; 5 Department of Experimental and Health Sciences, Pompeu Fabra University, 08002 Barcelona, Spain; 6 Rheumatology Department, Hospital del Mar, 08003 Barcelona, Spain; fojeda@psmar.cat (F.O.); jmonfort@psmar.cat (J.M.); 7 Cell Research on Inflammation and Cartilage, Programa de Recerca Clínica Tanslacional, Research Mar, 08003 Barcelona, Spain

**Keywords:** biopsychosocial approach, fibromyalgia, Fibromyalgia Impact Questionnaire (FIQ), Personality Assessment Inventory (PAI), personality traits, psychopathological profiles

## Abstract

**Background:** Fibromyalgia (FM) is a chronic condition characterized by widespread pain, fatigue, cognitive difficulties, and psychological symptoms. Patients often present distinct personality traits and psychopathological patterns associated with symptom severity. **Objective:** To examine psychopathological profiles in FM patients based on functional, physical–somatic, and emotional impairment domains, as well as on cumulative disease severity. **Materials and Methods:** A cross-sectional study was conducted with 70 women clinically diagnosed with FM at a specialized Fibromyalgia Unit. Psychological functioning was assessed using the Personality Assessment Inventory, and disease impact was measured with the Fibromyalgia Impact Questionnaire. Hierarchical cluster analyses were used to classify participants into mild and severe clusters across FIQ domains, and psychological profiles were compared. **Results:** Patients with severe functional impairment had more affective dysregulation (76.43 vs. 70.20, *p* < 0.01) and somatic complaints (85.57 vs. 79.76, *p* < 0.05) than those with mild impairment. The severe–physical cluster showed greater mood instability, somatization, and suicidal ideation (60.94 vs. 53.61, *p* < 0.05). The severe–emotional cluster had higher rates of major depression (85.71% vs. 64.28%) and persistent depressive disorder (76.19% vs. 70.61%, *p* < 0.05). Severe showed more emotional instability and somatization, distinguishing it from mild. Greater cumulative severity intensified depressive and somatic disorders. **Discussion:** Findings support FM’s biopsychosocial profile, where emotional distress may relate to psychological and physical symptoms, reinforcing the need for personalized, multidisciplinary care and comprehensive assessment.

## 1. Introduction

Fibromyalgia (FM) is a chronic syndrome characterized by widespread pain, fatigue, stiffness, sleep disturbances, and cognitive impairments (Bair & Krebs, 2020; Rizzi et al., 2017), although its etiology remains debated (Dizner-Golab et al., 2023). It is considered a central sensitization syndrome, involving dysfunction in pain-processing neural circuits (López-Ruiz et al., 2019; Pujol et al., 2014, 2022; Siracusa et al., 2021). Emotional distress exacerbates symptoms (Kocyigit & Akyol, 2022), hindering daily activities and work performance, significantly lowering well-being (Brown et al., 2023). This interaction underscores FM as both a physical and psychological condition. This duality is best captured by the biopsychosocial model, which conceptualizes illness as the result of dynamic interactions among biological, psychological, and social factors. Prevalence ranges from 2 to 8% worldwide (Siracusa et al., 2021; Berwick et al., 2022; Bair & Krebs, 2020) and reaches 2.4% in Spanish adults over 20 (Font Gayà et al., 2016). FM mainly affects women (4.2% vs. 0.2% in men) (Olfa et al., 2023; Kocyigit & Akyol, 2022), likely reflecting differences in pain and emotional processing. The highest prevalence (4.9%) is observed in ages 30–50 (Jurado-Priego et al., 2024), when functional demands exacerbate symptoms. Prevalence increases with age but declines after 80, likely due to symptom overlap or underreporting (Sarzi-Puttini et al., 2020).

Affective comorbidities are common in FM (37.7%), including hypervigilance, derealization, somatization, and elevated Cluster C (58.8%) or Cluster B (11.3%) traits (Dizner-Golab et al., 2023; Doreste et al., 2024; Romanov et al., 2023). FM symptom severity shapes psychopathological profiles: hypervigilance, suspicion, derealization, and impulsiveness are linked to fatigue and pain, while anxiety and depression relate to morning tiredness and stiffness (Attademo & Bernardini, 2018). Depressive disorders are the most prevalent diagnosis (43%) in FM, with major depression reaching 32% and persistent depression in 50–53% of cases (Doreste et al., 2025; Garcia-Fontanals et al., 2017; Munipalli et al., 2024). These affective disturbances impact core FM symptoms like pain and fatigue (Kim et al., 2023) and are often accompanied by cognitive deficits, including attentional, memory, and impulsivity issues (Aghbelaghi et al., 2024). Additionally, dissociative identity disorder appears in 16.6–18.2% of FM cases, linked to somatoform dissociation (48.5%), emotional dysregulation, and trauma (Doreste et al., 2024; Romeo et al., 2022a). FM patients also show social dysfunction resembling schizoid personality traits (1.9–22.2%) (Doreste et al., 2024; Romanov et al., 2023), with impulsivity associated with chronic pain and fatigue (Romanov et al., 2023).

Although personality disorders (PDs) are not a direct cause of FM, their comorbidity exacerbates emotional symptoms and complicates management. PD prevalence in FM ranges from 56.7% to 64.3% (Romanov et al., 2023; Doreste et al., 2024). Cluster C PDs—avoidant (3.8–28.8%), dependent (0–10%), and obsessive–compulsive (11.3–20%)—are predominant, though borderline (28.3%) and histrionic (1.9%) PDs from Cluster B are also observed (Romanov et al., 2023; Doreste et al., 2024). Type D personality, characterized by negative affect and social inhibition, correlates with higher FM severity (Garip et al., 2019). Personality traits like fear, rigidity, and anxiety reduce self-control and intensify anticipatory anxiety, often observed in more severe FM presentations (Garcia-Fontanals et al., 2017; Izquierdo-Alventosa et al., 2020). Dependent PD, in particular, is linked to increased fatigue, though this may be mitigated by autonomy and social support. Dependent, schizotypal, schizoid, and borderline personality traits predict FM severity, while Cluster B traits, though less prevalent, contribute to heightened pain and emotional distress (Izquierdo-Alventosa et al., 2020).

Given this complex interaction between physical and emotional symptoms, it is essential to further investigate FM’s psychological dimensions. FM is a heterogeneous disorder with highly variable symptom presentation, commonly classified according to physical characteristics, while psychological heterogeneity remains underexplored (Maurel et al., 2023; Sarzi-Puttini et al., 2020). Although no standardized subgrouping exists (Luciano et al., 2016), cluster analyses using self-reports have identified FM subtypes combining physical and psychopathological features. For instance, Keller et al. (2011) and de Souza et al. (2009) described subgroups differing in anxiety, depression, morning tiredness, fatigue, and joint stiffness, yet sharing common physical symptoms such as hyperalgesia. Similarly, Vincent et al. (2014) identified four subgroups based on overall symptom intensity, anxiety, and depression levels. Moreover, psychological distress, maladaptive cognitions, and poor coping strategies have been shown to amplify pain perception (Bucourt et al., 2019; Campos et al., 2021). Finally, SCL-90 profiles have linked somatization and obsessive traits to physical symptoms, while broader psychopathology is associated with complex comorbidities (Keller et al., 2011; Toussaint et al., 2015).

Although FM subtypes have been explored, most clustering studies focus narrowly on anxiety and depression, overlooking broader personality and psychopathological traits. Few examine how these vary across impact domains or cumulative severity, leaving psychological heterogeneity poorly defined and limiting tailored interventions. To address this gap—the limited exploration of psychological heterogeneity beyond anxiety and depression in FM—the present study used the Fibromyalgia Impact Questionnaire (FIQ) (Rivera & González, 2004) and the Personality Assessment Inventory (PAI) as core instruments (Burneo-Garcés et al., 2020). FIQ assesses FM’s impact across three domains: physical functioning, symptom severity (e.g., pain, fatigue, stiffness), and overall well-being, providing a detailed view of disability and quality of life (Keller et al., 2011; Maurel et al., 2023). The PAI offers a multidimensional evaluation of psychopathology aligned with DSM-5 criteria and has proven effective in chronic pain and FM to facilitate the identification of emotional and personality-related disturbances (Karlin et al., 2005). While both tools have been used independently, their combined application is novel in FM research. This integration allows for the classification of patients not only by functional severity but also by underlying psychological profiles, providing a richer and more clinically useful understanding of FM subtypes.

This study aimed to identify psychopathological profiles using the PAI, according to the type of FM impact in functional work, physical–somatic, and emotional domains as measured by the FIQ and cumulative severity. Given the multidimensional impact of fibromyalgia (FM) and its psychological complexity, can distinct psychopathological profiles be identified based on the functional, physical–somatic, and emotional domains of FM severity? We hypothesize that patients with severe functional impairment will exhibit higher emotional dysregulation and depressive symptoms (Ciuffini et al., 2023; Fischer-Jbali et al., 2021, 2022); those with pronounced physical–somatic impairment will be associated with heightened somatic complaints and mood instability (Creed, 2022; Hadlandsmyth et al., 2020); patients with severe emotional impairment will present a higher prevalence of depressive disorders and suicidality (Galvez-Sánchez et al., 2019; Levine & Horesh, 2020). Additionally, cumulative severity is expected to significantly intensify those psychopathological profiles, particularly emotional instability and somatization (Creed, 2022; Gonzalez et al., 2020). Identifying these profiles will enhance understanding of FM’s biopsychosocial complexity and support the development of personalized treatment approaches integrating both physical and psychological dimensions of the condition.

## 2. Materials and Methods

### 2.1. Eligibility Criteria

The research included females aged 18–65 diagnosed with FM based on American College of Rheumatology criteria (Wolfe, 1999). Inclusion criteria additionally required having stable pharmacological treatment, understanding the study requirements, and a commitment to compliance. Exclusion criteria encompassed the presence of other conditions explaining pain, inflammatory or rheumatic diseases, severe or unstable medical, endocrine, or neurological conditions, a history of neuropathic pain, acute psychotic disorders, substance abuse, and invalid scores on the FIQ and PAI validity scales, which could compromise data interpretation.

### 2.2. Participants

Patients were recruited from the Fibromyalgia Unit at Barcelona’s Hospital del Mar by senior rheumatologists (FO or JM) and a senior psychologist (JD) between January 2021 and June 2022 in clinical follow-up appointments. During this period, 136 female patients were diagnosed with FM, and 110 underwent eligibility assessments across consecutive clinical visits. A total of 40 patients either did not meet the study criteria or declined participation, yielding a final sample of seventy participants who completed both the FIQ and PAI questionnaires. Detailed sociodemographic and clinical characteristics can be found in Table 1.

### 2.3. Study Design and Procedure

We used a non-randomized, purposive sampling method to include all eligible participants from the study population. This observational, cross-sectional study involved female patients attending routine rheumatology appointments (FO and JM). After eligibility screening of inclusion/exclusion criteria, as well as confirmation of willingness to participate, patients were enrolled and provided informed consent. Psychological assessments, conducted by a senior clinical psychologist (AD), were scheduled within the same week and lasted up to 90 min to minimize response fatigue.

### 2.4. Instruments

The Personality Assessment Inventory (PAI) (Morey, 1991), in its Spanish adaptation (Burneo-Garcés et al., 2020), is a widely used, 344-item, self-reported measure designed to assess a broad spectrum of psychopathological symptoms and personality disorders. The PAI features 27 scales: 4 validity, 5 supplemental validity, 11 clinical, 5 treatment consideration, and 2 interpersonal, along with 31 clinically relevant subscales. This extensive range of scales enables the identification of various psychopathological patterns, covering 17 clinical syndromes and 11 personality disorders (Doreste et al., 2025; Ortiz-Tallo et al., 2011). Participants respond using a 4-point Likert-type scale (from 1—*not at all true* to 4—*very true*), and raw scores are converted to T-scores based on normative data from the Spanish population (Burneo-Garcés et al., 2020). Typically, a T-score above 61 indicates a moderate-to-high presence of psychopathological traits (Burneo-Garcés et al., 2020; Karlin et al., 2005; Morey & Alarcón, 2013). However, specific scales may require alternative cut-off scores to enhance diagnostic accuracy, as recommended in the PAI manual (Morey & Alarcón, 2013). The Spanish version of the PAI has demonstrated satisfactory psychometric properties, including internal consistency (Cronbach’s α = 0.82 overall; α = 0.78 in non-clinical samples and α = 0.83 in clinical populations), as well as content and convergent validity across diverse groups (Burneo-Garcés et al., 2020). In individuals with chronic pain, the PAI demonstrates acceptable internal consistency both for scale and subscale scores for assessing psychopathology patterns in chronic pain settings (Karlin et al., 2005).

The Fibromyalgia Impact Questionnaire (FIQ) (Burckhardt et al., 1991), in its Spanish version (Rivera & González, 2004), is a 10-item, self-administered tool that assesses the functional and overall impact of FM on daily living. It evaluates multiple aspects, including physical functioning, work-related limitations, and psychological well-being, offering a comprehensive picture of FM’s impact. Total score ranges from 0 to 100, with a higher score indicating greater disease impact and disability. The Spanish version has demonstrated good internal consistency (α = 0.81) and test–retest reliability over a 7-day period (with significant correlations from 0.52 for fatigue and 0.53 for pain to 0.91 for depression). It also provides evidence of validity based on its relationships with other variables, along with good sensitivity to changes over time (Monterde et al., 2004). Due to its ability to capture the multifaceted impact of FM, the FIQ is considered an essential instrument to quantify disability and guide treatment planning in both clinical and research settings.

### 2.5. Data Analysis

A descriptive analysis of sociodemographic and clinical features was conducted to delineate the characteristics of the entire study sample. All analyses were performed using IBM SPSS software (Version 21.0, IBM Corp, Armonk, NY, USA) for all analyses. Statistical significance was set at 5% and the sequence of the data analysis involved four main steps. Whenever applicable, 95% confidence intervals (CIs) were calculated to indicate the precision of group mean estimates:

**Cluster Analysis.** A hierarchical cluster analysis using Ward’s method with squared Euclidean distance was performed based on FIQ variables to classify patients into domains according to the severity of functional (Func), physical–somatic (Phys), and emotional (Emot) impairments. This method minimizes within-group variance, allowing for the identification of homogeneous and clinically meaningful domains. FIQ variables included in different domains were as follows: Func (work absence, physical function, and job performance), Phys (well-being, pain, fatigue, morning tiredness, and stiffness), and Emot (depression and anxiety). For each domain, a cluster analysis was conducted to classify participants into two severity levels: mild (M) and severe (S). Analysis resulted in six distinct clusters: Func-M and Func-S, Phys-M and Phys-S, as well as Emot-M and Emot-S.

**Discriminant function analysis** was conducted to validate the classifications obtained through cluster analysis and to identify the variables that best differentiated between severity levels within each domain, using Wilk’s Lambda as the test statistic. The assumption of homogeneity of covariance matrices was assessed using Box’s M test. Canonical correlation analysis was used to explore the relationship between the discriminant functions and the original FIQ variables. Finally, classification accuracy and cross-validation procedures were applied to evaluate the reliability of the group assignments made by the discriminant functions.

**Pairwise comparisons** were conducted to examine differences in psychological PAI scales, subscales, and clinical diagnostic categories between the severe and mild clusters within each domain (Func, Phys, and Emot). As the data violated the normality assumption (Shapiro–Wilk test), non-parametric Mann–Whitney U tests were used for these comparisons.

**Clinical Characterization**. Complementing these statistical comparisons, we also assessed the clinical patterns of each cluster by calculating the percentage of patients meeting criteria for positive psychological diagnoses. This analysis provided a more nuanced understanding of the psychopathological profiles associated with each severity level. Diagnoses were based on PAI scales and subscales, using a threshold score of 60 to indicate clinically significant symptomatology, in line with previous research (Doreste et al., 2024). In addition, published diagnostic criteria for dysthymia and comorbid dysthymia with major depression were applied to capture complex affective presentations not fully reflected by the standard PAI scoring (Doreste et al., 2025).

**Cumulative severity**. In the final step, we focused exclusively on the severe clusters to explore cumulative impact severity. Patients were grouped according to the number of severe clusters (Func-S, Phys-S, Emot-S) they belonged to, which are as follows: those in only one severe cluster (Single-S), in two (Dual-S), or in all three (Triple-S). Participants who did not belong to any severe cluster were classified as No-S. This classification captures the degree to which patients experience multidimensional impairment, which may reflect increasing clinical complexity or greater treatment needs. Illustrative clinical examples by cumulative severity group: Single-S: Patient A reports chronic somatic complaints (e.g., fatigue, headaches) but shows no significant emotional or functional impairment. Dual-S: Patient B presents with both physical symptoms and emotional dysregulation (e.g., anxiety, irritability), which moderately interfere with daily functioning. Triple-S: Patient C experiences severe impairment across physical, emotional, and functional domains, including depressive episodes, chronic pain, and marked social withdrawal, suggesting high clinical complexity and elevated treatment needs. Based on this classification, a non-parametric Kruskal–Wallis test was used to compare PAI scales, subscales, and positive psychological diagnoses across cumulative severity groups.

## 3. Results

**Figure 1:**
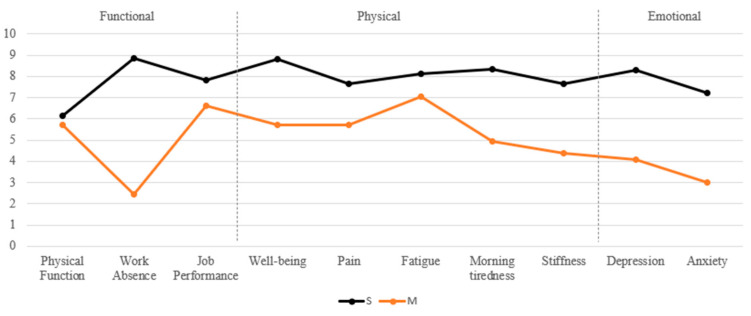
**S:** severe cluster, **M:** mild cluster.

**Descriptive and cluster analysis.** Patients (N = 70) were analyzed across the three impact domains—Func, Phys, and Emot—and further subdivided into mild (M) and severe (S) impairment levels: Func-M (n = 49, 70.0%) vs. Func-S (n = 21, 30.0%), Phys-M (n = 18, 25.7%) vs. Phys-S (n = 52, 74.3%), and Emot-M (n = 28, 40.0%) vs. Emot-S (n = 42, 60.0%). Descriptive analysis of each FIQ impact domain across M and S clusters can be seen in Figure 1.

**Discriminant functional analysis** validated the FIQ-based classification of domains and clusters with functional, physical–somatic, and emotional impairment (Table 2 and Table 3).

In the functional domain, three predictors were included as follows: physical function, work absence, and job performance. Significant differences were found for work absence and job performance, while physical function was not a significant contributor. A single discriminant function explained 100% of the variance with a strong canonical correlation. Work absence was the strongest predictor, followed by job performance; physical function had negligible impact. The model achieved 94.3% overall accuracy, correctly classifying 95.9% of Func-M and 90.5% of Func-S cases.

In the physical–somatic domain, predictors were well-being, pain, fatigue, morning tiredness, and stiffness. All variables showed significant differences. A single discriminant function again explained 100% of the variance with a strong canonical correlation. Morning tiredness was the most influential predictor, followed by stiffness, well-being, and pain. Fatigue showed a lower, inverse contribution. Classification accuracy reached 100%, correctly classifying all Phys-M and Phys-S cases.

In the emotional domain, depression and anxiety were used as predictors. Both differed significantly between severity groups. A single discriminant function accounted for 100% of the variance, with strong canonical correlation. Anxiety was the strongest predictor, followed by depression. The model achieved 97.1% accuracy, correctly classifying 97.6% of Emot-M and 96.4% of Emot-S participants.

**Figure 2:**
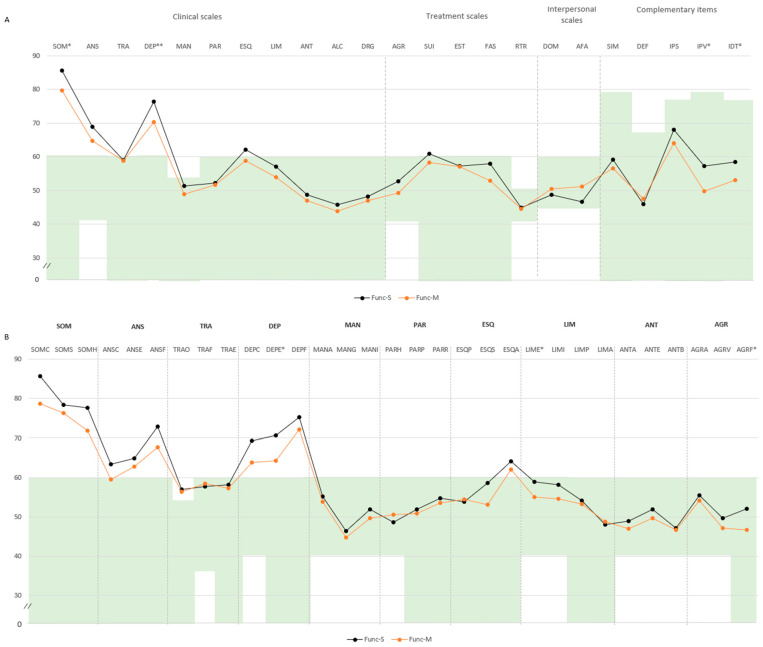
Comparison of PAI scale and subscale T-scores between mild and severe clusters for the functional impairment domain. *Note.* (**A**) Comparison between clusters in PAI scales and complementary items; (**B**) comparison between clusters in PAI subscales. The green zone indicates the ranges of normality, according to the psychometric criteria of the PAI. **SOM:** Somatic Complaints; **ANS:** Anxiety; **TRA:** Disorders Related to Anxiety; **DEP:** Depression; **MAN:** Mania; **PAR:** Paranoia; **ESQ:** Schizophrenia; **LIM:** Limit Traits; **ANT:** Antisocial Traits; **ALC:** Problems with alcohol; **DRG:** Problems with drugs; **AGR:** Aggression: **SUI:** Suicidal Ideation; **EST:** Stress; **FAS:** Lack of social support; **RTR:** Refusal to treatment; **DOM:** Dominance; **AFA:** Affability; **SIM:** Simulation Index; **DEF:** Defensiveness Index; **IPS:** Potential Suicide Index; **IPV:** Potential index of violence; **IDT:** Treatment Difficulty Index; **Func-S:** Fibromyalgia functional severe cluster; **Func-M:** Fibromyalgia functional mild cluster; (**B**) **SOM-C:** Conversion; **SOM-S:** Somatization; **SOM-H:** Hypochondria; **ANS-C:** Cognitive; **ANS-E:** Emotional; **ANS-F:** Physiological; **TRA-O:** Obsessive–compulsive; **TRA-F:** Phobias; **TRA-E:** Posttraumatic Stress; **DEP-C:** Cognitive; **DEP-E:** Emotional; **DEP-F:** Physiological; **MAN-A:** Activity level; **MAN-G:** Grandeur; **MAN-I:** Irritability; **PAR-H:** Hypervigilance; **PAR-P:** Persecution; **PAR-R:** Resentment; **ESQ-P:** Experiences. Psychotics; **ESQ-S:** Social Indifference; **ESQ-A:** Alteration of the Thought; **LIM-E:** Emotional instability; **LIMI:** Alteration of identity; **LIM-P:** Problematic Interpersonal Relationships; **LIM-A:** Self-aggression; **ANT-A:** Antisocial Behaviors; **ANT-E:** Egocentrism; **ANT-B:** Search for sensations; **AGR-A:** Attitude aggressive; **AGR-V:** Verbal aggression; **AGR-F:** Physical assaults; tatistically significant differences between groups: * *p* < 0.05; ** *p* < 0.01.

**Pairwise comparison.** In the functional domain, participants classified as Func-S showed higher scores across several psychopathological domains compared to Func-M. Specifically, significant differences were found in negative impression (M = 71.4, SD = 14.8 vs. M = 62.9, SD = 14.3), somatic disorders (SOM: M = 85.5, SD = 7.7 vs. M = 79.7, SD = 10.0), and depression (DEP: M = 76.4, SD = 10.4 vs. M = 70.2, SD = 9.2), with all mean scores exceeding the defined cut-off point of 60. Subscale differences included higher scores in health concerns (SOM-H: M = 77.6, SD = 11.0 vs. M = 71.8, SD = 10.0) and emotional depression (DEP-E: M = 70.7, SD = 11.3 vs. M = 64.2, SD = 11.7), both above the cut-off point. Additional differences were noted in emotional instability (M = 58.9, SD = 9.5 vs. M = 55.0, SD = 9.1) and physical aggression (M = 52.0, SD = 11.4 vs. M = 46.6, SD = 5.5). Higher scores in violence index (M = 57.2, SD = 14.7 vs. M = 49.8, SD = 8.4) and treatment difficulties (M = 58.4, SD = 10.6 vs. M = 53.0, SD = 8.6) indicated increased aggressiveness potential and treatment difficulties (Figure 2A,B).

**Figure 3:**
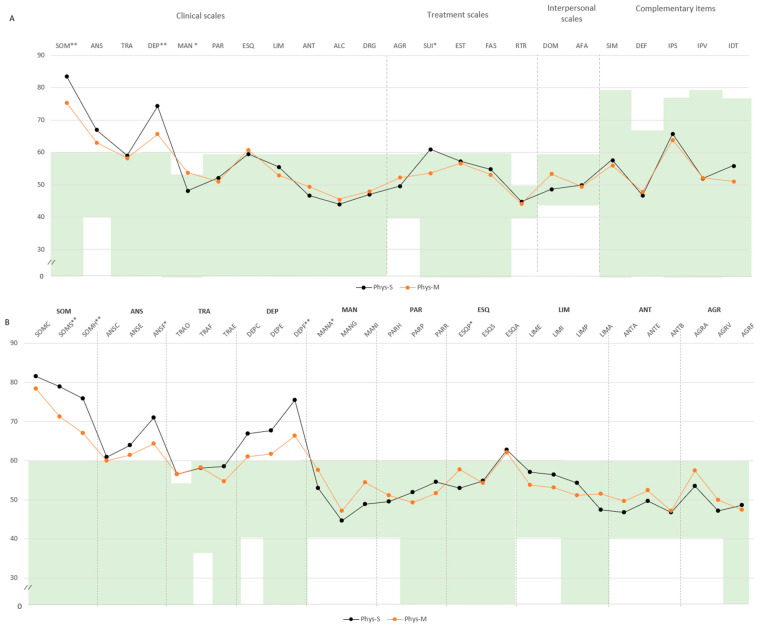
Comparison of PAI scale and subscale T-scores between mild and severe clusters for the physical impairment domain. Note: (**A**) Comparison between clusters in PAI scales and complementary items; (**B**) comparison between clusters in PAI subscales. The green zone indicates the ranges of normality, according to the psychometric criteria of the PAI. **SOM:** Somatic Complaints; **ANS:** Anxiety; **TRA:** Disorders Related to Anxiety; **DEP:** Depression; **MAN:** Mania; **PAR:** Paranoia; **ESQ:** Schizophrenia; **LIM:** Limit Traits; **ANT:** Antisocial Traits; **ALC:** Problems with alcohol; **DRG:** Problems with drugs; **AGR:** Aggression: **SUI:** Suicidal Ideation; **EST:** Stress; **FAS:** Lack of social support; **RTR:** Refusal to treatment; **DOM:** Dominance; **AFA:** Affability; **SIM:** Simulation Index; **DEF:** Defensiveness Index; **IPS:** Potential Suicide Index; **IPV:** Potential index of violence; **IDT:** Treatment Difficulty Index; **Phys-S:** Fibromyalgia physical severe cluster; **Phys-M:** Fibromyalgia physical mild cluster; (**B**). Note: **SOM-C:** Conversion; **SOM-S:** Somatization; **SOM-H:** Hypochondria; **ANS-C:** Cognitive; **ANS-E:** Emotional; **ANS-F:** Physiological; **TRA-O:** Obsessive–compulsive; **TRA-F:** Phobias; **TRA-E:** Posttraumatic Stress; **DEP-C:** Cognitive; **DEP-E:** Emotional; **DEP-F:** Physiological; **MAN-A:** Activity level; **MAN-G:** Grandeur; **MAN-I:** Irritability; **PAR-H:** Hypervigilance; **PAR-P:** Persecution; **PAR-R:** Resentment; **ESQ-P:** Experiences. Psychotics; **ESQ-S:** Social Indifference; **ESQ-A:** Alteration of the Thought; **LIM-E:** Emotional instability; **LIMI:** Alteration of identity; **LIM-P:** Problematic Interpersonal Relationships; **LIM-A:** Self-aggression; **ANT-A:** Antisocial Behaviors; **ANT-E:** Egocentrism; **ANT-B:** Search for sensations; **AGR-A:** Attitude aggressive; **AGR-V:** Verbal aggression; **AGR-F:** Physical assaults; statistically significant differences between groups: * *p* < 0.05; ** *p* < 0.01.

In the physical–somatic domain, Phys-S individuals showed higher scores in SOM (M = 83.6, SD = 9.0 vs. M = 75.4, SD = 9.3) and DEP (M = 74.2, SD = 8.2 vs. M = 65.6, SD = 11.9), both exceeding the defined cut-off point. Additional differences emerged in mania (MAN: M = 48.1, SD = 9.7 vs. M = 53.7, SD = 8.4) and suicidal ideation (SUI: M = 60.9, SD = 16.1 vs. M = 53.6, SD = 17.0). Subscale comparisons revealed higher scores in somatization (SOM-S: M = 78.9, SD = 7.6 vs. M = 71.2, SD = 7.6), SOM-H (M = 75.8, SD = 10.2 vs. M = 66.9, SD = 8.9), physical anxiety (ANS-F: M = 70.9, SD = 11.2 vs. M = 64.3, SD = 11.2), and physical depression (DEP-F: M = 75.4, SD = 6.6 vs. M = 66.3, SD = 9.1), all above the clinical threshold (Figure 3A,B).

**Figure 4:**
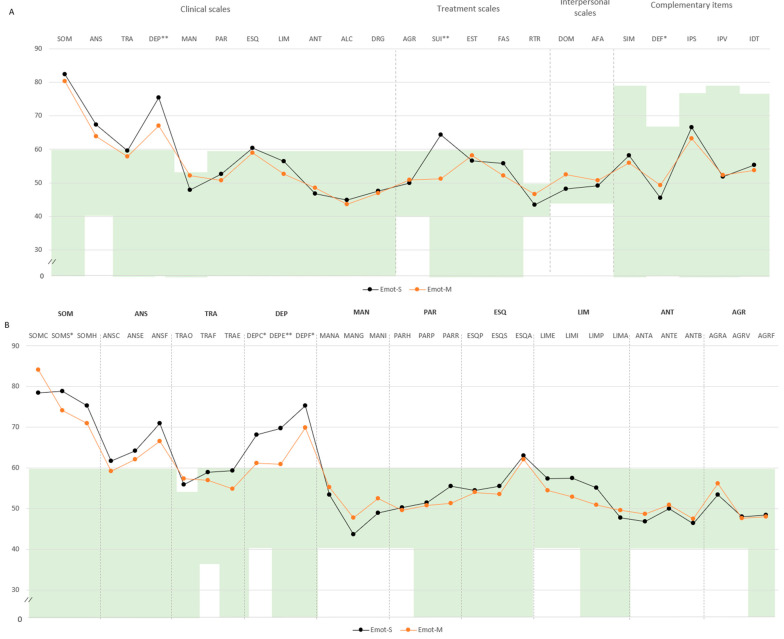
Comparison of PAI scale and subscale T-scores between mild and severe clusters for the physical impairment domain. Note. (**A**) Comparison between clusters in PAI scales and complementary items; (**B**) comparison between clusters in PAI subscales. The green zone indicates the ranges of normality, according to the psychometric criteria of the PAI. **SOM:** Somatic Complaints; **ANS:** Anxiety; **TRA:** Disorders Related to Anxiety; **DEP:** Depression; **MAN:** Mania; **PAR:** Paranoia; **ESQ:** Schizophrenia; **LIM:** Limit Traits; **ANT:** Antisocial Traits; **ALC:** Problems with alcohol; **DRG:** Problems with drugs; **AGR:** Aggression: **SUI:** Suicidal Ideation; **EST:** Stress; **FAS:** Lack of social support; **RTR:** Refusal to treatment; **DOM:** Dominance; **AFA:** Affability; **SIM:** Simulation Index; **DEF:** Defensiveness Index; **IPS:** Potential Suicide Index; **IPV:** Potential index of violence; **IDT:** Treatment Difficulty Index; **Emot-S:** Fibromyalgia emotional severe cluster; **Emot-M:** Fibromyalgia emotional mild cluster; (**B**). Note: **SOM-C:** Conversion; **SOM-S:** Somatization; **SOM-H:** Hypochondria; **ANS-C:** Cognitive; **ANS-E:** Emotional; **ANS-F:** Physiological; **TRA-O:** Obsessive–compulsive; **TRA-F:** Phobias; **TRA-E:** Posttraumatic Stress; **DEP-C:** Cognitive; **DEP-E:** Emotional; **DEP-F:** Physiological; **MAN-A:** Activity level; **MAN-G:** Grandeur; **MAN-I:** Irritability; **PAR-H:** Hypervigilance; **PAR-P:** Persecution; **PAR-R:** Resentment; **ESQ-P:** Experiences. Psychotics; **ESQ-S:** Social Indifference; **ESQ-A:** Alteration of the Thought; **LIM-E:** Emotional instability; **LIMI:** Alteration of identity; **LIM-P:** Problematic Interpersonal Relationships; **LIM-A:** Self-aggression; **ANT-A:** Antisocial Behaviors; **ANT-E:** Egocentrism; **ANT-B:** Search for sensations; **AGR-A:** Attitude aggressive; **AGR-V:** Verbal aggression; **AGR-F:** Physical assaults; statistically significant differences between groups: * *p* < 0.05; ** *p* < 0.01.

In the emotional domain, Emot-S participants had higher levels of psychopathology. Specifically, DEP (M = 75.4, SD = 8.5 vs. M = 67.0, SD = 9.8), SUI (M = 64.2, SD = 17.5 vs. M = 51.2, SD = 11.3), and SOM-S (M = 78.8, SD = 8.0 vs. M = 74.0, SD = 7.9) exceeded the threshold. Subscale analyses indicated greater scores in cognitive depression (DEP-C: M = 68.2, SD = 10.9 vs. M = 61.2, SD = 12.6), DEP-E (M = 69.6, SD = 11.9 vs. M = 60.8, SD = 10.0), and DEP-F (M = 75.2, SD = 7.1 vs. M = 69.8, SD = 8.9). Lower defensiveness was also noted (M = 45.4, SD = 7.2 vs. M = 49.3, SD = 7.5) (Figure 4A,B).

**Figure 5:**
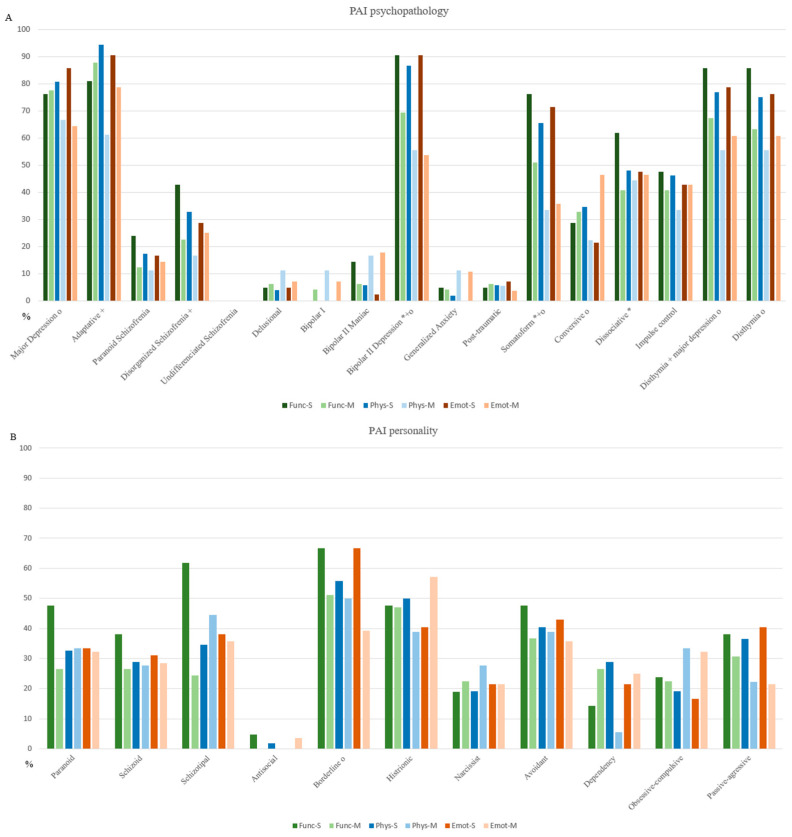
Comparison of PAI scale and subscale T-scores between mild and severe clusters for the functional, physical and emotional impairment domain. *Note.* (**A**) PAI psychopathology profiles; (**B**) PAI personality profiles. **Func:** Functional subgroup, **Phys:** Physical subgroup, **Emot:** Emotional subgroup, **S**: severe cluster, and **M:** mild cluster. Pairwise statistical significances at *p* < 0.05: *: Func-S vs. Func-M, +: Phys-S vs. Phys-M, and **ο:** Emot-S vs. Emot-M.

Regarding the clinical diagnostic categories, Func-S showed higher and clinically significant scores in bipolar II depressive disorder (68.57 ± 7.76 vs. 63.78 ± 7.44), somatic disorder (65.17 ± 7.16 vs. 61.87 ± 6.36), and dissociative disorder (62.84 ± 8.04 vs. 58.40 ± 8.86). Phys-S exhibited elevated scores mainly in adaptive disorder (63.84 ± 3.04 vs. 60.73 ± 2.99) and bipolar II depressive disorder (66.77 ± 6.84 vs. 60.72 ± 8.83). Emot-S presented higher scores in major depression (66.18 ± 6.53 vs. 61.00 ± 7.79), bipolar II depressive disorder (67.63 ± 6.85 vs. 61.60 ± 7.85), and persistent depressive disorder (65.31 ± 6.81 vs. 60.52 ± 7.73) with major depression (63.36 ± 4.65 vs. 59.87 ± 5.46) (Figure 5A,B).

**Clinical characterization.** The proportion of patients meeting the diagnostic criteria was calculated to compare the prevalence of psychopathological diagnoses between the severe (S) and mild (M) clusters within each domain (Func, Phys, and Emot). Statistical significance is shown in Figure 5A,B.

Bipolar II depression emerged as the most severe and pervasive condition across all domains. A diagnosis was considered positive when the corresponding T-score exceeded the clinical threshold of 60. In the Func domain, 90.47% of Func-S participants met this threshold, compared to 69.39% in Func-M. Similarly, 86.54% of Phys-S and 55.56% of Phys-M participants scored above the threshold. In the Emot domain, the difference was even more pronounced, with 90.47% of Emot-S versus 53.57% of Emot-M showing clinically significant scores. These findings suggest that bipolar II depression exerts a broader and more intense impact among individuals in the severe clusters.

Other psychopathological conditions also showed widespread impairment across domains. Both somatic disorder and dysthymia with major depression were markedly prevalent. Somatic disorder showed a higher positive percentage in functional (76.19% Func-S vs. 51.02% Func-M), physical (65.38% Phys-S vs. 33.33% Phys-M), and emotional domains (71.43% Emot-S vs. 35.71% Emot-M). Similarly, dysthymia with major depression percentages were substantial across functional (85.71% Func-S vs. 67.35% Func-M), physical (76.92% Phys-S vs. 55.56% Phys-M), and emotional domains (78.57% Emot-S vs. 60.71% Emot-M). Adaptative disorder showed the highest prevalence in the physical domain, with 94.23% in Phys-S decreasing to 61.11% in Phys-M, while borderline disorder presents substantial emotional distress (66.67% Emot-S vs. 39.29% Emot-M), suggesting a more moderate impact in milder cases. Diagnoses such as dissociative, obsessive–compulsive, and passive-aggressive disorder were more commonly observed in the Func-S. Dissociative prevalence dropped from 61.90% in Func-S to 40.82% in Func-M, while obsessive–compulsive remained low across both severity levels (23.81% Func-S, 22.45% Func-M). Conversely, conversive, impulsive, and dysthymia disorders predominantly impacted emotional functioning. Notably, disthymia showed 76.19% of Emot-S patients meeting the diagnostic threshold. The findings suggest that bipolar II depressive, somatic disorder, and dysthymia with major depression are the most functionally and emotionally debilitating conditions across all domains, while adaptative disorder and bipolar II depressive were most strongly associated with physical symptoms.

**Cumulative Severity:** Participants were distributed across four cumulative severity groups: No-S (n = 11; 15.7%), Single-S (n = 14; 20.0%), Double-S (n = 35; 50.0%), and Triple-S (n = 10; 14.3%). In the Single-S group, Phys-S was the most prevalent severity cluster with 71.4% of cases (n = 10). In the Double-S group, Phys-S (n = 10, 91.4%) and Emot-S (n = 29, 82.9%) emerged as the predominant cluster.

**Figure A1:**
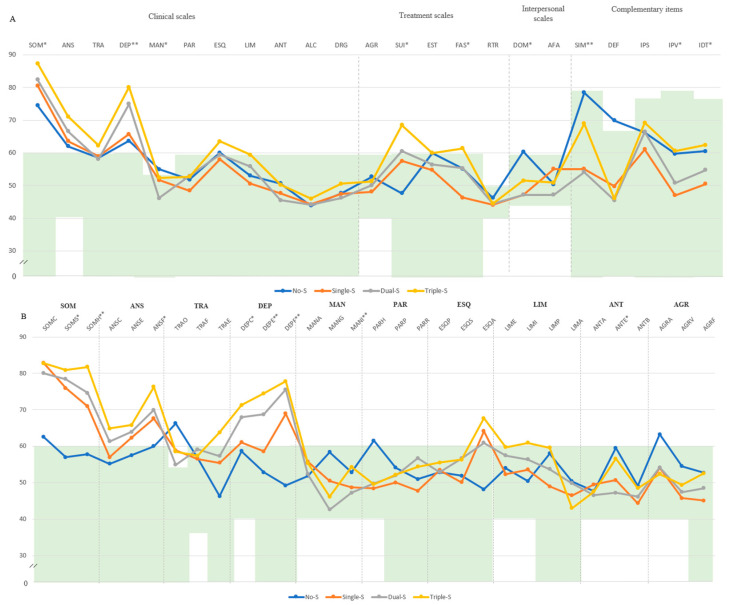
PAI psychopathology scales and subscales according to cumulative severity groups by FIQ. Note. (**A**) Comparison between groups in PAI scales and complementary items; (**B**) comparison between groups in PAI subscales. The green zone indicates the ranges of normality, according to the psychometric criteria of the PAI. **SOM:** Somatic Complaints; **ANS:** Anxiety; **TRA:** Disorders Related to Anxiety; **DEP:** Depression; **MAN:** Mania; **PAR:** Paranoia; **ESQ:** Schizophrenia; **LIM:** Limit Traits; **ANT:** Antisocial Traits; **ALC:** Problems with alcohol; **DRG:** Problems with drugs; **AGR:** Aggression: **SUI:** Suicidal Ideation; **EST:** Stress; **FAS:** Lack of social support; **RTR:** Refusal to treatment; **DOM:** Dominance; **AFA:** Affability; **SIM:** Simulation Index; **DEF:** Defensiveness Index; **IPS:** Potential Suicide Index; **IPV:** Potential index of violence; **IDT:** Treatment Difficulty Index; **No-S:** patients not classified as severe in any domain; **Single-S:** patients classified as severe in one domain; **Dual-S**: patients classified as severe in two domains; **Triple-S:** patients classified as severe in all three domains; (**B**). Note: **SOM-C:** Conversion; **SOM-S:** Somatization; **SOM-H:** Hypochondria; **ANS-C:** Cognitive; **ANS-E:** Emotional; **ANS-F:** Physiological; **TRA-O:** Obsessive–compulsive; **TRA-F:** Phobias; **TRA-E:** Posttraumatic Stress; **DEP-C:** Cognitive; **DEP-E:** Emotional; **DEP-F:** Physiological; **MAN-A:** Activity level; **MAN-G:** Grandeur; **MAN-I:** Irritability; **PAR-H:** Hypervigilance; **PAR-P:** Persecution; **PAR-R:** Resentment; **ESQ-P:** Experiences. Psychotics; **ESQ-S:** Social Indifference; **ESQ-A:** Alteration of the Thought; **LIM-E:** Emotional instability; **LIMI:** Alteration of identity; **LIM-P:** Problematic Interpersonal Relationships; **LIM-A:** Self-aggression; **ANT-A:** Antisocial Behaviors; **ANT-E:** Egocentrism; **ANT-B:** Search for sensations; **AGR-A:** Attitude aggressive; **AGR-V:** Verbal aggression; **AGR-F:** Physical assaults; statistically significant differences between all groups: * *p* < 0.05; ** *p* < 0.01.

**Figure A2:**
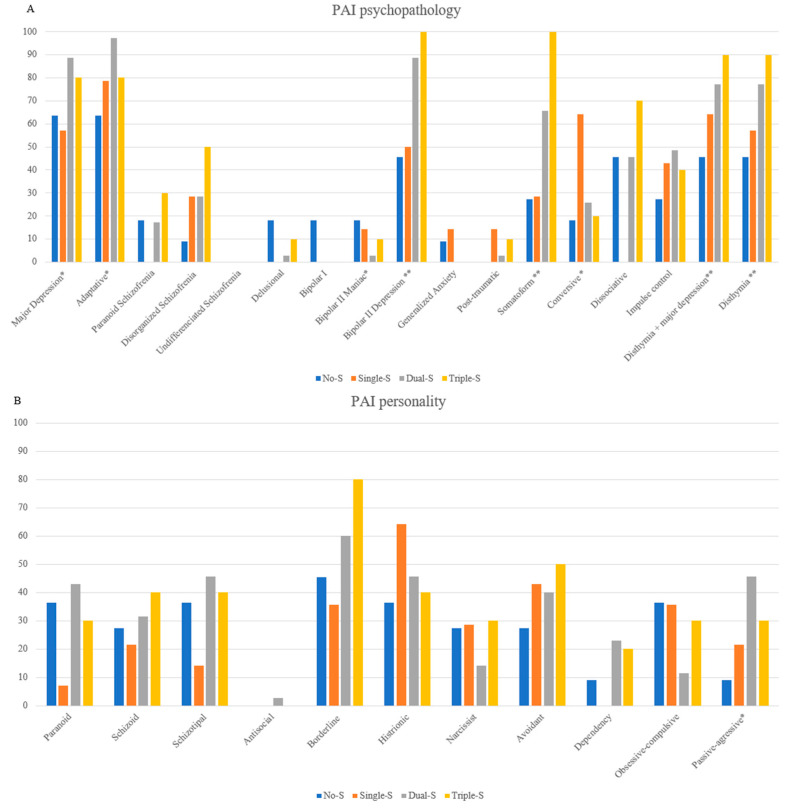
(**A**). Comparison of PAI-based psychopathology (top) and personality disorder (down) profiles between cumulative severity groups by FIQ. *Note.* (**A**) PAI psychopathology profiles. (**B**) PAI personality diagnoses. **No-S:** fibromyalgia absence of cluster severity; **Single-S:** fibromyalgia one cluster severity; **Dual-S:** fibromyalgia two cluster severities; **Triple-M:** fibromyalgia three cluster severities. Statistical significances: * *p* < 0.05; ** *p* < 0.01.

Moving to group comparison, in the Triple-S group, participants displayed notably higher scores compared to those in the Double-S group, particularly for SOM (M = 87.0, SD = 8.44 vs. M = 82.7, SD = 8.9), DEP: M = 80.10, SD = 7.78 vs. M = 74.94, SD = 7.47), and SUI (M = 68.50, SD = 19.02 vs. M = 60.57, SD = 16.41). In contrast, within the No-S group, MAN (M = 55.00, SD = 9.75) and DOM (M = 60.36, SD = 12.71) presented scores outside the normal range. The graded increase in severity across groups was clearly evident, with the Triple-S group consistently surpassing Double-S and Single-S in symptom burden. This pattern was particularly pronounced in subscales such as SOM-S (M = 80.90, SD = 6.29), SOM-H (M = 81.80, SD = 9.94), ANS-F (M = 76.30, SD = 8.24), DEP-C (M = 71.30, SD = 10.89), DEP-E (M = 74.50, SD = 10.77), and DEP-F (M = 77.80, SD = 7.58), all significantly elevated compared to the No-S group (seen Figure A1). Additionally, Triple-S was associated with the highest prevalence percentage of bipolar II depression (100%), somatoform disorder (100%), dysthymia (90%), and persistent depression with major depression (90%). On the other hand, Dual-S showed the highest prevalence for major depression (88.57%), adaptative disorder (97.14%), and passive-aggressive traits (45.71%) while Single-S had the highest prevalence for conversive disorder (64.29%) (see Figure A2). Descriptive analysis of the results can be found in Appendix A Table A1.

## 4. Discussion

The findings of this study show distinct psychological profiles according to the type and severity of functional, physical, and emotional impairment. Individuals with Func-S exhibited higher levels of negative impression, somatic complaints, emotional depressive symptoms, emotional instability, physical aggression, and treatment resistance, along with increased diagnoses of depressive and somatic disorders. In the Phys-S cluster, somatic and depressive symptoms, suicidal ideation, manic traits (especially in relation to activity levels), and psychotic experiences predominated, which is rarely emphasized in the FM literature. These participants also exhibited higher rates of adaptive functioning difficulties, disorganized schizophrenia traits, somatic disorders, and bipolar II-related depressive and manic episodes. In the Emot-S cluster, the clinical profile was characterized by cognitive, emotional, and physiological depressive symptoms, suicidal ideation, somatization, and lower defensiveness, with a notable increase in diagnoses of depressive disorders, somatic disorders, persistent depression with or without major depression. Finally, cumulative severity was associated with greater psychological impairment. The Triple-S group showed the highest scores in somatization, depression, negative impression, and physical anxiety, representing a distinct clinical profile. This cumulative severity was also accompanied by a higher number of clinical diagnoses like depression, somatoform and persistent depression with and without major depression.

In line with the elevated emotional instability and depressive symptoms observed in the Func-S group, severe functional impairment was associated with cognitive overload and executive dysfunctions, including attention, memory, and decision-making difficulties (Castel et al., 2021; Pallanti et al., 2021), which contribute to daily limitations beyond physical symptoms (Jacobsen et al., 2023). Occupational difficulties were central, as work absence had a greater psychological toll than general physical limitations, reflecting the profound impact of occupational identity loss on self-esteem and well-being (Berk, 2020; Chang et al., 2019; Van Alboom et al., 2021). Social withdrawal and interpersonal strain further exacerbated emotional distress and isolation, perpetuating a vicious cycle of functional decline and increased suicidality risk (Gil-Ugidos et al., 2021; Levine & Horesh, 2020; Martínez et al., 2021). Additionally, barriers to treatment adherence—both emotional and practical—compounded this complex clinical picture (Bulu et al., 2023; Campos et al., 2021; Sarzi-Puttini et al., 2021). Our findings suggest that such profiles may benefit from integrative interventions combining work reintegration strategies, cognitive rehabilitation, structured social support, and meaningful alternatives to occupational tasks (Castel et al., 2021; Gil-Ugidos et al., 2021; Van Alboom et al., 2021).

Consistent with the elevated somatic, depressive, and psychotic symptoms observed in the Phys-S group, severe physical impairment was linked to intensified pain perception, mood instability, and psychotic-like experiences, suggesting sensory and autonomic dysregulation as key mechanisms (Islam et al., 2022; Nadal-Nicolás et al., 2020). Central sensitization likely underlies the excessive somatic symptoms (Bhargava & Goldin, 2025; Gelonch et al., 2018; Siracusa et al., 2021), while autonomic dysfunctions such as orthostatic intolerance (Alsiri et al., 2022) and temperature dysregulation (Islam et al., 2022) further compromise physical capacity (Taylor et al., 2021). Immune dysregulation (García-Domínguez, 2025) and chronic inflammation have also been associated with heightened pain, fatigue, and depressive symptoms, underscoring the interplay between physical and psychiatric manifestations (Meade & Garvey, 2022). Behaviorally, pain-related activity avoidance due to kinesiophobia contributed to deconditioning and functional decline (Serrat et al., 2021; İnal et al., 2020; Robbins et al., 1990), highlighting the need for targeted movement therapies (Bravo et al., 2019; Serrat et al., 2021). Psychotic-like features, including disorganized schizophrenia traits (Almulla et al., 2020), may reflect stress-related cognitive-perceptual disturbances (Bulu et al., 2023) rather than primary psychosis (Zinchuk et al., 2025). The presence of both depressive and manic traits, often linked to bipolar II-related episodes (Bortolato et al., 2016; Dell’Osso et al., 2009; Gota et al., 2017), calls for integrated interventions targeting mood regulation (Kudlow et al., 2015) alongside physical rehabilitation (Araújo & DeSantana, 2019; Serrat et al., 2021).

Individuals in the Emot-S group exhibited pervasive depressive symptoms (Wolfe, 1999; Yuan et al., 2015), stress hypersensitivity (Lahat-Birka et al., 2024), and maladaptive coping strategies (Conversano et al., 2019), reinforcing the biopsychosocial model of FM (Wolfe & Rasker, 2021). Diagnoses of persistent depression and major depression, alongside high rates of suicidal ideation, suggest that emotional distress may be explained by previous findings on serotonergic and dopaminergic dysregulation, mood instability, motivational deficits (Doreste et al., 2025; Estévez-López et al., 2019; Sedda et al., 2025), and pain sensitization (Ansari et al., 2021). Furthermore, maladaptive cognitive–emotional processes such as cognitive distortions (Vittersø et al., 2022), negative bias (Zetterman et al., 2021), catastrophizing (Izquierdo-Alventosa et al., 2020), and somatic amplification (Vittersø et al., 2022) maintain a psychosomatic loop in which emotional distress both stems from and worsens FM symptoms (Araújo & DeSantana, 2019). Autonomic overactivation (Goldway et al., 2022) and exaggerated stress responses (Arslan & Ünal Çevik, 2022) further heighten pain perception and psychological burden. Social and interpersonal difficulties, including defensiveness (Berk, 2020; Romeo et al., 2022b) and low social support (Cagliyan Turk et al., 2024; Galvez-Sánchez & del Paso, 2020; Gonzalez et al., 2020; Pacheco-Barrios et al., 2024), further exacerbate emotional suffering and isolation. Given this complex interplay, interventions should extend beyond conventional mood treatments (Castel et al., 2021; Hirsch et al., 2021) to incorporate emotional regulation training (Trucharte et al., 2020), relational and social support approaches (Puşuroğlu et al., 2023), and strategies targeting the neurobiological mechanisms underlying FM’s emotional dimension (Al Sharie et al., 2024). Across all domains, patients with severe impairment consistently exhibited elevated depressive symptoms, somatic complaints, and suicidal ideation (Adawi et al., 2021), suggesting shared psychopathological patterns regardless of the primary area of impact. This cumulative pattern suggests that greater accumulation of affected domains—functional, physical–somatic, and emotional—is associated with more severe psychopathological profiles, underscoring the clinical relevance of multidimensional assessment to guide individualized and multidisciplinary treatment strategies in fibromyalgia.

The convergence of severe functional, physical, and emotional impairments in the Triple-S group underscores that fibromyalgia severity is not merely additive but exponentially compounded. This cumulative impact reflects a convergence of biopsychosocial vulnerabilities that intensify core symptoms such as somatization, depression, and anxiety, while personality traits remain relatively stable. Rather than representing isolated symptom domains, the co-occurrence suggests a synergistic deterioration driven by shared psychological mechanisms—learned helplessness contributing to functional decline, pain catastrophizing and hypervigilance amplifying physical symptoms, and emotional dysregulation reinforcing psychological burden. Emotional impairment is further reinforced by affective dysregulation, chronic stress, and maladaptive coping. These findings underscore the need for integrated, multidisciplinary interventions targeting cognitive, emotional, and behavioral factors alongside physical health to improve outcomes (Hirsch et al., 2021).

This study has limitations that should be considered. The cross-sectional design prevents establishing causality between psychological factors and FM symptoms. Self-reported measures, including the PAI and the FIQ, may introduce biases such as social desirability and recall errors. Also, the use of the original version of the Fibromyalgia Impact Questionnaire (FIQ), which, while validated, does not include certain symptom domains covered by more recent versions such as the FIQ-R. This may limit the comprehensiveness of symptom assessment in the current study. The sample size, while sufficient for analysis, may limit generalizability. Additionally, unmeasured confounders like medication use, comorbid conditions, and coping strategies could influence results. Another limitation is the absence of a control group, which limits our ability to determine whether the identified psychological profiles are specific to fibromyalgia or reflect general patterns observed in chronic pain populations. This study is limited by its exclusive use of a Spanish sample, reliance on self-report instruments (PAI and FIQ), and the absence of control for psychiatric comorbidities, which may affect the generalizability and specificity of the findings. Furthermore, the inclusion of only female participants restricts the generalizability of findings to male patients, who may exhibit different symptom patterns or psychological responses to fibromyalgia.

The stratification of FM patients into functional, physical–somatic, emotional, and cumulative severity profiles offers promising avenues for clinical application. In triage, early identification of patients in the Triple-S or Emot-S profiles could prioritize those at highest risk for poor outcomes, suicidality, or treatment resistance. In routine care, psychological profiles could guide more targeted interventions—e.g., combining cognitive rehabilitation for Func-S, pain neuroscience education for Phys-S, and emotion regulation therapy for Emot-S. Moreover, these clusters could serve as stratification variables in clinical trials to examine differential treatment responses or to test tailored intervention packages. Future research should explore the predictive validity and clinical utility of this typology in prospective, real-world settings. Longitudinal studies with larger, more diverse samples and other objective assessments are also needed to confirm these findings.

## 5. Conclusions

In conclusion, to our knowledge, this is the first study to integrate the Fibromyalgia Impact Questionnaire (FIQ) with the Personality Assessment Inventory (PAI), offering a novel multidimensional approach to classify fibromyalgia patients based on both functional impact and underlying psychopathological profiles. This study confirmed our hypotheses by identifying distinct psychological profiles based on the type and severity of functional, physical, and emotional impairment in individuals with fibromyalgia (FM), with important clinical implications. Each group—Func-S, Phys-S, Emot-S, and Triple-S—exhibited unique patterns of symptoms and psychiatric diagnoses, all reflecting a complex interplay of biopsychosocial factors. Cumulative severity, as seen in the Triple-S group, was associated with greater psychological and clinical impairment, suggesting that integrated treatment approaches addressing physical, emotional, and cognitive dimensions may be beneficial, particularly for patients with more severe or complex profiles.

## Figures and Tables

**Table 1:** Descriptive data of sociodemographic and clinical variables of the study sample (N = 70).

Descriptive Data M (SD)	
Age (years)	48.86 (8.32)
Fibromyalgia tender points (0–18)	17.21 (1.39)
Diagnosis Time in Years	6.70 (6.71)
Symptom Onset Time (Years)	14.94 (11.22)
**Educational attainment (%)**	
Primary Studies	10.0
Secondary Studies	11.4
Bachelor	17.1
Professional Studies	32.8
University	28.5
**Stable medication regime (%)**	
Analgesic (NSAIDs and/or Opioids)	68.6
Anti-inflammatory	58.2
Antidepressant	73.1
**Type of antidepressant (%)**	
ISRS	31.3
Dual	17.9
Tricyclic	25.3
Benzodiazepine	37.3
**Type of benzodiazepine (%)**	
Short-acting	14.9
Intermediate-acting	4.4
Long-acting	17.9
**FM symptoms (FIQ ^1^** **)**	
FIQ global score (0–100) M (SD)	66.82 (13.67)
**Physical Functioning Dimension**	
Physical function (0–10) M (SD)	5.85 (2.25)
**Global Impact Dimension**	
Well-being (0–10) M (SD)	8.04 (2.61)
Work absence (0–10) M (SD)	4.33 (3.59)
**Symptoms Dimension**	
Job performance (0–10) M (SD)	6.99 (1.95)
Pain (0–10) M (SD)	7.14 (1.64)
Fatigue (0–10) M (SD)	7.84 (1.37)
Morning tiredness (0–10) M (SD)	7.46 (2.08)
Stiffness (0–10) M (SD)	6.80 (2.46)
Anxiety (0–10) M (SD)	6.60 (2.65)
Depression (0–10) M (SD)	5.54 (2.90)

^1^ **FIQ:** Fibromyalgia Impact Questionnaire. N = 70. M = mean. SD = standard deviation.

**Table 2:** Domains discriminant function analysis.

Subgroup	Wilk’s Lambda	Chi-Square	Canonical Correlation	Eigenvalue	M-Box	*p*-Value (M-Box)
Functional	0.248	92.756	0.867	3.034	9.265	0.192
Physical–somatic	0.277	84.053	0.850	2.608	52.919	<0.001
Emotional	0.294	82.129	0.841	2.407	42.126	<0.001

**Table 3:** Variables discriminant function analysis results.

Variable	Wilk’s Lambda	F-Value	*p*-Value	Standard Coefficient	Structure Coefficient
**Functional domain**					
Physical Function	0.992	0.57	0.454	−0.364	0.052
Work Absence	0.277	177.26	<0.001	1.129	0.927
Job Performance	0.923	5.71	0.020	−0.165	0.166
**Physical–somatic domain**					
Well-being	0.713	27.35	<0.001	0.831	0.393
Pain	0.738	24.16	<0.001	0.664	0.369
Fatigue	0.884	8.94	0.004	−0.378	0.224
Morning tiredness	0.489	71.07	<0.001	0.564	0.633
Stiffness	0.664	34.42	<0.001	0.354	0.440
**Emotional domain**					
Depression	0.479	73.91	<0.001	0.583	0.672
Anxiety	0.385	108.83	<0.001	0.746	0.815

## Data Availability

The data presented in this study are available on request from the corresponding author due to privacy restrictions.

## Appendix A

**Table A1:** Descriptive analysis of results in each domain, cluster and cumulative severity groups. **Note. FIQ:** Fibromyalgia Impact Questionnaire. **PAI:** Personality Assessment Inventory. **Func-S:** fibromyalgia functional severe cluster; **Func-M:** fibromyalgia functional mild cluster; **Phys-S:** fibromyalgia physical severe cluster; **Phys-M:** fibromyalgia physical mild cluster; **Emot-S:** fibromyalgia emotional severe cluster; **Emot-M:** fibromyalgia emotional mild cluster; **No-S:** fibromyalgia absence of cluster severity; **Single-S:** fibromyalgia one cluster severity; **Dual-S:** fibromyalgia two cluster severities; **Triple-M:** fibromyalgia three cluster severities.

Impact Domain (FIQ)	Cluster	PAI Psychological Characteristics	Most Prevalent Diagnosis by PAI
**Functional**	Func-M	Milder emotional distress and fewer physical symptom concerns	Major depression (77.55%) and adaptative disorder (87.75%). Borderline personality disorder (51%).
	Func-S	Increased sensitivity to symptoms, mood variability, and treatment engagement difficulties	Bipolar II depression (90.47%), Somatoform disorder (76.19%), dissociative disorder (61.90%), dysthymia (85.71%), and dysthymia with Major Depression (85.71%). Schizotypal (61.90%) and borderline (66.66%) personality disorder.
**Physical–somatic**	Phys-M	Higher energy levels with relatively stable mood and fewer emotional symptoms (depressive and anxiety features)	Bipolar II depressive (55.55%), borderline (50%), narcissist (27.77%), and avoidant (38.88%) personality disorder.
	Phys-S	Heightened sensitivity to bodily symptoms and mood-related challenges with suicidal ideation	Major depression (80.76%), adaptative (94.23%), bipolar II depression (86%). Somatoform (65.38%), dysthymia (75%), and dysthymia with major depression (76.92%).
**Emotional**	Emot-M	Functional physical responses to stress, dominance or directive interpersonal style with limited emotional distress and low suicidal ideation	Conversive disorder (46.42%) and histrionic personality disorder (57.14%).
	Emot-S	Pronounced emotional (depression and anxiety) and physical distress (somatization), with reduced emotional resilience or lowered coping barriers	Major depression (85.71%), adaptative disorder (90.47%), bipolar II depression (90.47%), dysthymia (76.19%), and dysthymia with major depression (78.57%).
**Cumulative**	No-S	Cautious self-presentation or defensive coping style and oppositional attitudes under stress	Obsessive–compulsive personality disorder (36.36%).
	Single-S	Stable interpersonal functioning with adequate support and few treatment challenges, as well as a supportive psychosocial environment	Psychogenic disorder (64.29%) and obsessive–compulsive personality disorder (35.71%).
	Dual-S	Stable activity levels and mood regulation, with no signs of elevated impulsivity or energy dysregulation	Major depression (88.57%) and adaptative disorder (97.14%). Paranoid (42.86%), schizotypal (45.71%), and passive-aggressive personality disorder (45.71%).
	Triple-S	Severe multidimensional distress marked by intense symptom preoccupation, emotional suffering with suicidal ideation, as well as heightened physical and psychological sensitivity	Bipolar II depressive (100%), somatoform (100%), dysthymia (90%), and dysthymia with major depression (90%). Borderline (80%) and avoidant personality disorder (50%).
